# Compression properties and constitutive model of short glass fiber reinforced poly-ether-ether-ketone (PEEK)

**DOI:** 10.1038/s41598-023-46078-z

**Published:** 2023-11-06

**Authors:** Ruijie Zhang, Li Chen, Kai Xie, Kun Liu, Zhilin Wu

**Affiliations:** School of Mechanical Engineering, Nanjing University of Science and Technology, Nanjing, 210094 Jiangsu China

**Keywords:** Composites, Mechanical properties

## Abstract

To analyze the deformation behavior of short glass fiber-reinforced poly-ether-ether-ketone (SGFR-PEEK) under various conditions through numerical simulations, it is crucial to construct a constitutive model that can describe its stress–strain behavior over a wide range of strain rates and temperatures. In this study, quasi-static compression tests were conducted on SGFR-PEEK composites with varying mass fractions, and dynamic tests were performed using a Split Hopkinson Pressure Bar to acquire the material's compressive stress–strain response under quasi-static and dynamic conditions. The results indicate that, under compression, the yield stress of SGFR-PEEK composites increases with an augmentation in glass fiber content, rises with increasing strain rate, and decreases with elevated temperature. Based on experimental findings, a modified Johnson–Cook constitutive model was established to characterize the mechanical performance of SGFR-PEEK. In comparison to the traditional Johnson–Cook intrinsic structure model, the modified model takes into account the glass fiber mass fraction as comprehensively as possible and better predicts the material's flow behavior at high strain rates. Finally, this modified constitutive model was implemented in the ABAQUS software using the user-defined subroutine VUMAT to simulate the compression behavior of SGFR-PEEK composites under different loading conditions, and the model was validated. This research provides valuable insights for the practical application of SGFR-PEEK composites in engineering.

## Introduction

Polyether ether ketone (PEEK) is a semi-crystalline thermoplastic polymer with excellent mechanical properties, such as high strength, high modulus, and high toughness, as well as outstanding thermal stability and chemical inertness. It is widely used in the fields of medicine, automotive manufacturing, aerospace, and others^1,2^. To address the issue that a single PEEK material is unable to meet various application requirements, targeted approaches such as fiber reinforcement, particle filling, surface modification, and blending with other polymers can be employed to improve its performance^3–6^. Among these methods, short fiber reinforced PEEK (SFR-PEEK) is a moderately reinforced system that bridges the gap between unenhanced PEEK and continuous fiber-reinforced PEEK. Compared to unreinforced PEEK, SFR-PEEK exhibits superior stiffness and strength. Moreover, its processing technology has seen significant advancements, contributing to extensive prospects for applications^7,8^.

To ensure that SFR-PEEK can be better applied in engineering practice, it is necessary to obtain the material's mechanical properties under different working conditions. Typically, universal material testing machines and Hopkinson bar devices are used to test the material's quasi-static and dynamic performance. Rae et al.^9^ tested the compressive performance of PEEK at strain rates of 10^–4^ to 3000 s^−1^ and temperatures ranging from -85 to 200°C, and found that the mechanical properties of the material were strongly correlated with temperature and strain rate. Additionally, researchers have also studied topics such as notch sensitivity, fatigue, mechanical impact resistance, and the effect of microstructural inclusions on the fatigue life of PEEK^10–13^. For fiber-reinforced PEEK materials, Li et al.^14^ obtained the mechanical properties of carbon fiber-reinforced PEEK (CF/PEEK) through a series of experiments, and compared to pure PEEK, the material exhibited higher hardness, tensile, and compressive strength after carbon fiber reinforcement. Similarly, Zhang et al.^15^ compared the reinforcing effect of various fibers on PEEK matrix and found that carbon fibers, glass fibers, and TiO_2_ can effectively improve the tensile strength of the matrix. Gao et al.^16^ further analyzed the mechanical properties of different components and types of fiber-reinforced PEEK materials by controlling the fiber type and content. These studies are of great help in understanding the mechanical properties of PEEK composite materials, and based on the experimental results, more accurate constitutive models can be constructed to evaluate the influence of strain rate and temperature on material performance.

As a classic phenomenological constitutive model, the Johnson–Cook model for hot-drawn plasticity can consider the effects of strain rate and temperature on plastic stress^17^. Due to its decoupling term and fewer constitutive parameters, the amount of experimental data required to fit its parameters is relatively small, and it is also easier to implement in commercial simulation software. In recent years, quite a few studies based on the Johnson–Cook model and its improved models have achieved good results in describing the mechanical properties of metals and non-metals^18–20^. For PEEK materials, Chen et al.^21,22^ tested the compression performance of the material under strain rates ranging from 0.01 to 1 s^−1^ and temperatures ranging from 23 to 150 °C, and, combined with the experimental data from literature^9^, established an improved Johnson–Cook model to describe the mechanical behavior of PEEK, especially pointing out that the yield stress is inversely proportional to temperature. Literature^18^ and literature^19^ established constitutive models for the material, and, based on custom subroutines, reproduced the studied material's stress–strain in finite element calculation software, successfully simulating the material's response under corresponding application scenarios. This quantitative description of the material's mechanical properties helps to simulate the material's response under different working conditions, making it easier to optimize engineering designs for the material's usage conditions.

In this study, the quasi-static mechanical properties of SGFR-PEEK at different temperatures were tested using a general-purpose materials testing machine with a thermostat box, and the dynamic mechanical properties at different strain rates were obtained using a Hopkinson press bar. The sensitivity of the compressive properties of the material to temperature and strain rate was analyzed, and a modified Johnson–Cook constitutive model taking into account the fiber content was proposed to describe the relationship between stress, strain rate, and temperature. In addition, a user-defined material subroutine, VUMAT, was developed to implement the improved constitutive model in Abaqus^®^/Explicit software. The correctness of the improved constitutive model was verified by comparing the experimental results with the simulation results.

## Experiments

### Materials and specimens

The SGFR-PEEK composites were produced by Nanjing Comptech^®^ Composites Corporation (Nanjing, China). These composites consist of fibers with an approximate diameter of 10 μm and lengths ranging from 50 to 200 μm. The melting temperatures (*T*_*m*_) of various weight fractions of SGFR-PEEK composites were determined through Differential Scanning Calorimetry (DSC) analysis, as summarized in Table 1. It can be seen that with an increase in the glass fiber content, the *T*_*m*_ of SGFR-PEEK composites slightly decreases, consistent with the test data in reference^23^.

**Table 1:** Melting temperature of SGFR-PEEK composites.

	PEEK + 10%SGF	PEEK + 20%SGF	PEEK + 30%SGF
*T*_*m*_ (°C)	342.15	341.78	340.83

### Quasi-static compression tests

**Figure 1:**
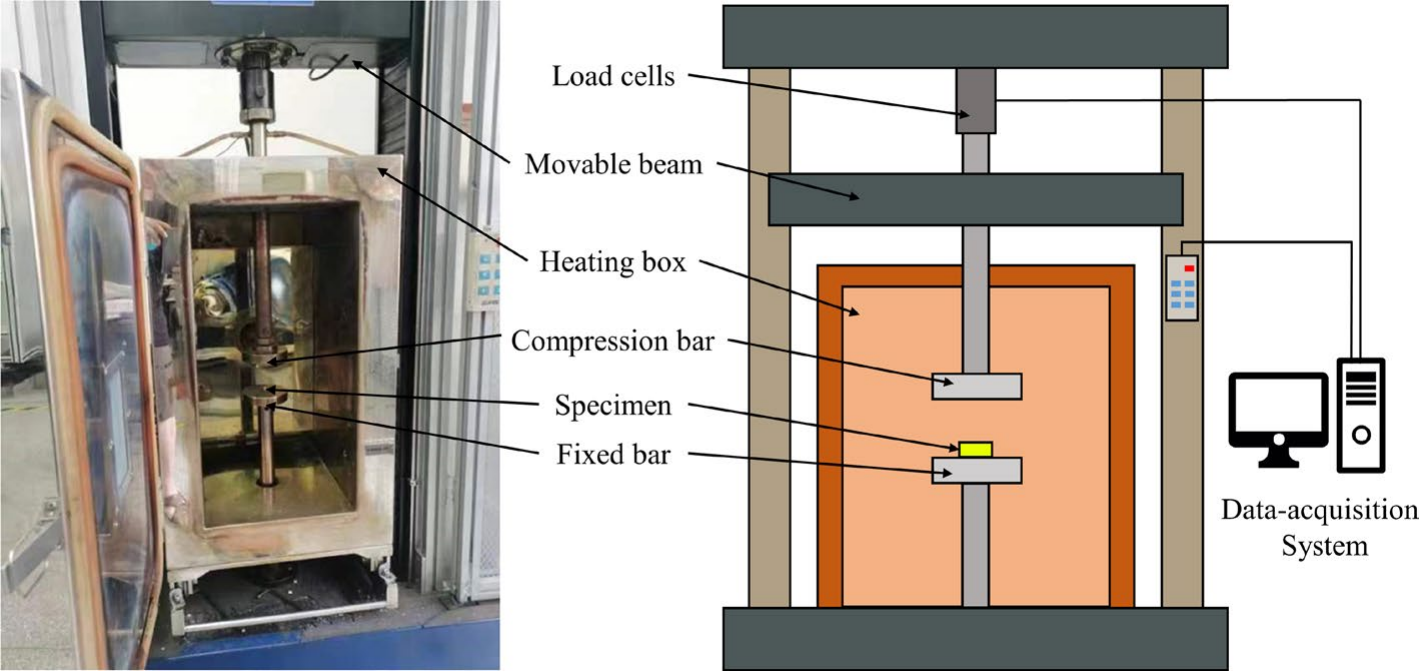
Quasi-static compression test.

The specimens for quasi-static tests are cylindrical, following the standard GB/T1041-92, with dimensions of ∅10 mm × 15 mm. The experiments were conducted on an electrical universal testing machine, as depicted in Fig. 1. The compression experiments were conducted on an electrically-powered universal testing machine, as depicted in Fig. 1. This universal testing machine is equipped with a heating box designed for evaluating the mechanical properties of materials under different temperature conditions.

The mechanical properties of SGFR-PEEK were tested at room temperature (23 °C) with strain rates of 0.001 s^−1^, 0.01 s^−1^, 0.1 s^−1^, and at elevated temperatures of 50 °C, 100 °C, 150 °C, and 200 °C with a strain rate of 0.001 s^−1^. Prior to the variable temperature tests, the specimens were heated and held for 30 min to ensure temperature uniformity.

In the experiments, the engineering stress-engineering strain (${\sigma }_{e}-{\varepsilon }_{e}$) curve could be directly obtained through the data acquisition system, while the true stress-true strain curve (${\sigma }_{t}-{\varepsilon }_{t}$) was calculated using Eq. (1).

1 $$ \left\{ \begin{gathered} \sigma_{t} = \sigma_{e} (1 - \varepsilon_{e} ) \hfill \\ \varepsilon_{t} = - \ln (1 - \varepsilon_{e} ) \hfill \\ \end{gathered} \right. $$

### Dynamic compression tests

**Figure 2:**
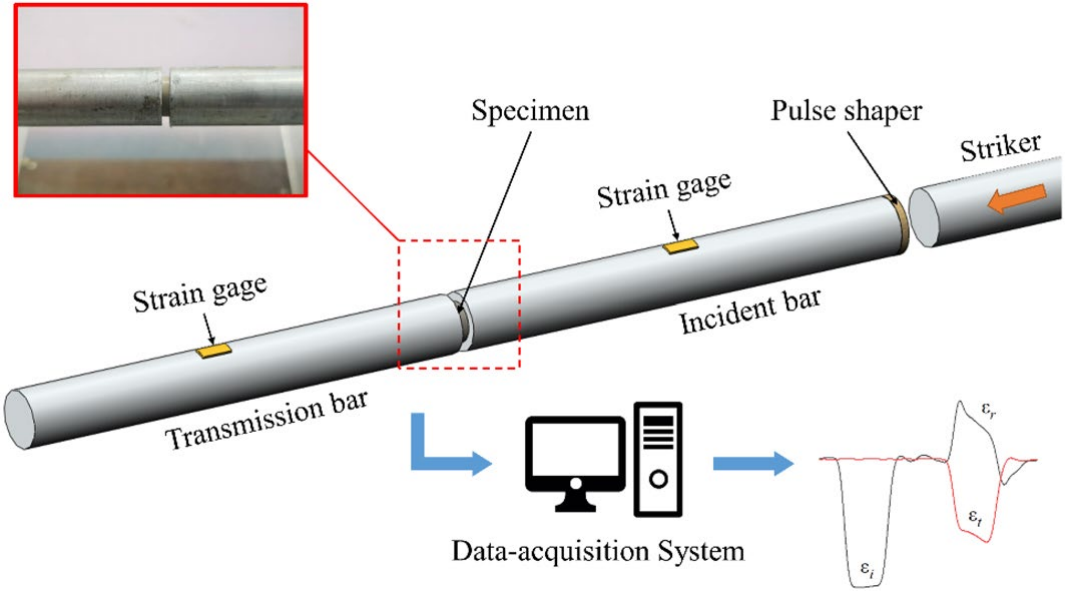
Dynamic compression test.

The dynamic compression tests are performed on split Hopkinson compression bar apparatus, as shown in Fig. 2, to test the mechanical properties of the material at room temperature (23 °C) with strain rates of 800 s^−1^, 1400 s^−1^, 2000 s^−1^, and 2500 s^−1^. The experimental compression rod is a 14.5 mm diameter hard aluminum alloy (LC4) with the elastic modulus of 72 GPa, yield strength of 490 MPa, and density of 2800 kg/m^3^. The lengths of the incident and transmission rods are 1000 mm, and the lengths of the impact and absorption rods are 300 mm.

When conducting dynamic compression tests, achieving a state of stress equilibrium within the specimen requires at least one transmission and reflection of stress waves. To ensure the precision of the experiments, the specimen dimensions were chosen as ∅10 mm × 2 mm in thickness to expedite the time required for the specimen to reach a state of stress equilibrium^24,25^. Additionally, a layer of Vaseline was applied as a lubricant at the points where the specimen contacts the rods to minimize errors resulting from frictional effects.

For data acquisition, the incident and reflected signals were measured using strain gauges affixed to the incident rod, while the transmitted signals were measured using strain gauges mounted on the transmission rod. These strain gauges were positioned at a fixed distance of 500 mm from the specimen. The data acquisition system operated at a sampling frequency of 10 MHz, facilitating real-time visualization of all pulse signals through LabVIEW software.

**Figure 3:**
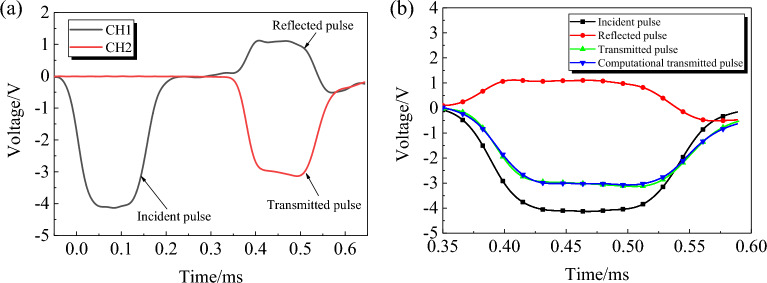
Typical test waveform (**a**) and dynamic stress equilibrium (**b**).

The SHPB test is based on two basic assumptions: that the stress wave in the rod propagates as a one-dimensional elastic wave and that the specimen is uniformly deformed. On the basis of the test waveform, the engineering strain, engineering stress, and strain rate of the specimen can be obtained from Eq. (2)^26–29^.

2 $$ \left\{ \begin{gathered} \sigma (t) = \frac{A}{{2A_{0} }}E(\varepsilon_{i} (t) + \varepsilon_{r} (t) + \varepsilon_{t} (t)) \hfill \\ \dot{\varepsilon }(t) = \frac{{C_{0} }}{{L_{0} }}(\varepsilon_{i} (t) + \varepsilon_{r} (t) + \varepsilon_{t} (t)) \hfill \\ \varepsilon (t) = \int_{0}^{t} {\frac{{C_{0} }}{{L_{0} }}(\varepsilon_{i} (t) + \varepsilon_{r} (t) + \varepsilon_{t} (t))} {\text{ d}}t \hfill \\ \end{gathered} \right. $$

where ${\varepsilon }_{i}$, ${\varepsilon }_{r}$, ${\varepsilon }_{t}$ are the incident, reflected, and transmitted waveforms with time, respectively, *L*_0_ and *A*_0_ are the thickness and cross-sectional area of the specimen, *A* is the cross-sectional area of the compression bar, and *C*_0_ is the wave speed in the compression bar. A typical set of waveforms in the test is given in Fig. 3.

The true stress-true strain curve of the dynamic compression test is also obtained by Eq. (1).

## Results and discussion

### Experimental results

**Figure 4:**
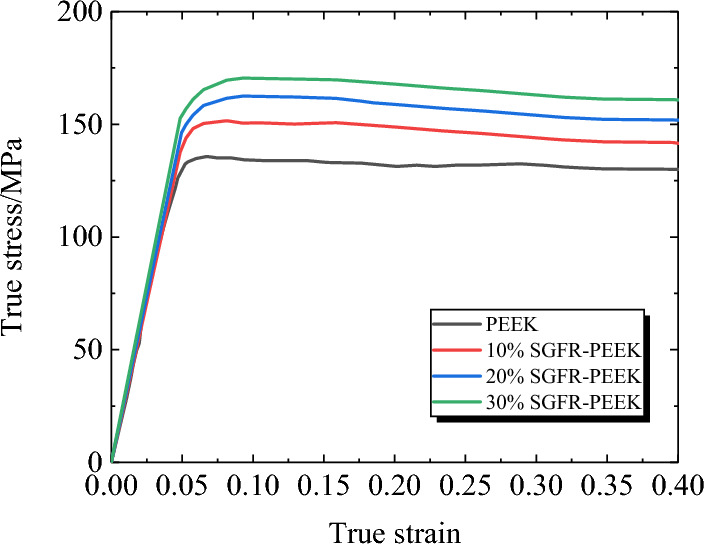
True stress–strain curves of PEEK^9^ and SGFR-PEEK (*T* = 23°C, $\dot{\varepsilon }$=0.001s^−1^).

Figure 4 presents the true stress–strain curves of the PEEK matrix and SGFR-PEEK composites with different weight fractions under room temperature (23°C) and quasi-static ($\dot{\varepsilon }$=0.001s^−1^) conditions. It can be observed that the SGFR-PEEK composites exhibit an elastic modulus close to that of the PEEK matrix, with a noticeable increase in yield strength. Additionally, as the mass fraction of short glass fibers increases, the yield strength of the composites also rises. In comparison to the yield strength of the PEEK matrix at 134 MPa, the SGFR-PEEK composite material with a 30% weight fraction demonstrates a yield strength of 152 MPa, representing a 13.4% improvement.

**Figure 5:**
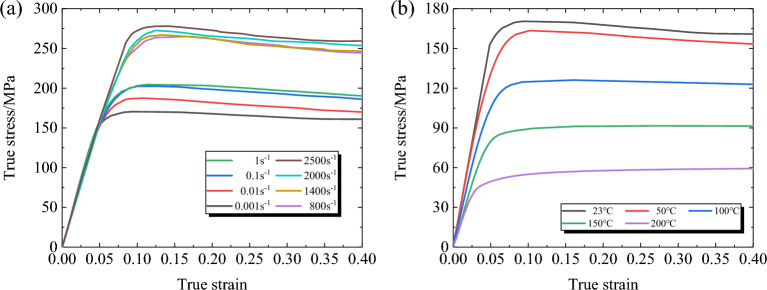
True stress–strain curves under different deformation conditions. (**a**) *T* = 20°C; (**b**) $\dot{\varepsilon }$=0.001s^−1^).

Figure 5 illustrates the true stress–strain curves of 30% SGFR-PEEK composites under various testing conditions. Combining this with Fig. 4, it is observed that the stress–strain curves of both the PEEK matrix and SGFR-PEEK composites conform to the four-stage classification outlined in reference^30^: In the first stage (work hardening stage), the work hardening rate is higher than the softening rate caused by dynamic recovery, resulting in a sharp increase in stress at the beginning of micro-strain deformation, followed by an increase in the rate of stress reduction. The second stage (transition stage) is characterized by the competition between dynamic recovery-induced work hardening and softening phenomena and dynamic recrystallization. Additionally, the shear stress continues to increase, but at a decreasing rate. In the third stage (softening stage), the stress drops sharply, which is related to dynamic recovery, dynamic recrystallization, and other factors. Finally, in the fourth stage (steady stage), the stress tends to stabilize when a new balance between softening and hardening is achieved.

Table 2 presents additional insights into the yield strength of both the PEEK matrix and SGFR-PEEK composites under diverse loading conditions. Notably, the yield strength of SGFR-PEEK composites is influenced significantly by fiber content. Under equivalent conditions, the yield stress increases with higher strain rates and decreases with elevated temperatures. For instance, with a 30% fiber content, the material exhibits a 54.6% enhancement in yield strength at 2500 s^−1^ compared to 0.001 s^−1^ at 23 °C. Conversely, under quasi-static loading, the material's yield strength at 200 °C is 77.2% lower than that at 23 °C. These findings underscore the pronounced influence of temperature and strain rate on the mechanical properties of both the PEEK matrix and SGFR-PEEK composites.

**Table 2:** Yield stress of PEEK^9^ and SGFR-PEEK under diverse loading conditions.

*T*/°C	$\dot{\varepsilon }$/s^−1^	PEEK	10%SGFR-PEEK	20%SGFR-PEEK	30%SGFR-PEEK
23	0.001	134.18	138.75	146.28	152.08
	0.01	141.24	144.89	150.30	160.26
	0.1	147.75	152.14	157.16	166.70
	1	156.43	161.83	171.63	182.18
	100	184.12			
	800				222.62
	1000		210.75	218.48	
	1200		214.25		
	1400			224.09	228.52
	2000		218.63	226.87	232.5
	2500		223.99		235.54
	2900	219.92			
50	0.001	119.53	123.60	130.31	135.47
100		93.75	96.94	102.20	106.25
150		60.94	63.01	66.43	69.16
200		30.47	31.50	33.22	34.53

### Constitutive equations for SGFR + PEEK composites

As an image-only intrinsic structure model, the Johnson–Cook intrinsic structure model has been commonly used for numerical calculations in explosions, impacts, and stamping. In recent years, the JC model and the modified JC model were used to describe the stress changes in PEEK at different temperatures and strain rates with good results^21,22,31^.

Based on the true stress-true strain curves obtained from quasi-static and dynamic tests, each parameter in the JC model can be determined.

The traditional phenomenological JC model may be expressed as:

3 $$ \sigma = \left( {A + B\varepsilon_{p}^{n} } \right)\left( {1 + C\ln \frac{{\dot{\varepsilon }}}{{\dot{\varepsilon }_{ref} }}} \right)\left[ {1 - T^{*m} } \right] $$

where $\upsigma$ $\upsigma$ is the equivalent flow stress, ${\varepsilon }_{p}$ is the equivalent plastic strain, *A* is the yield stress at the reference temperature and reference strain rate, *B* is the coefficient of strain hardening, *n* is the strain hardening exponent, *C* and *m* are the material constants which represent the coefficient of strain rate hardening and thermal softening exponent, $\dot{\varepsilon }$ is the strain rate, while ${\dot{\varepsilon }}_{ref}$ is the reference strain rate.

T^*^ is the homologous temperature and is expressed as:

4 $$ T^{*} = \frac{{T - T_{ref} }}{{T_{m} - T_{ref} }} $$

where *T* is temperature. *T*_*ref*_ is the reference temperature. Tm is the melting temperature.

### *Constitutive equations for 30%SGFR* + *PEEK*

To obtain the parameters of the classical Johnson–Cook intrinsic model, 0.001s^−1^ was used as the reference strain rate (${\dot{\varepsilon }}_{ref}$) and 20 °C as the reference temperature (*T*_*ref*_). A is the yield stress of the material at the reference strain rate and reference temperature, which can be determined as 152.08 MPa. *T*_*m*_ is the melting temperature, 340.83 °C.

At the reference strain rate and reference temperature, Eq. (3) can be written as

5 $$ \sigma = A + B\varepsilon_{p}^{n} $$

Taking the logarithm of both sides of the equation yields,

6 $$ \ln (\sigma - A) = n\ln \varepsilon_{p} + \ln B $$

the values of *n* and *B* can be determined as 0.079 and 14.585 MPa.

At the reference strain rate, Eq. (5) can be written as:

7 $$ \frac{\sigma }{{A + B\varepsilon_{p}^{n} }} = 1 - \left( {\frac{{T - T_{ref} }}{{T_{m} - T_{ref} }}} \right)^{m} $$

the *C* value obtained using the straight-line fit is 0.0412.

Taking the logarithm of both sides, taking the logarithm of both sides of Eq. (8),

8 $$ \ln \left[ {1 - \frac{\sigma }{{A + B\varepsilon^{n} }}} \right] = m\ln \left( {\frac{{T - T_{ref} }}{{T_{m} - T_{ref} }}} \right) $$

The value of *m* can be determined from the slope of the average fitted line as 0.962.

The classical Johnson–Cook model with all parameters determined by the above calculations can be written as:

9 $$ \sigma = \left( {152.08 + 14.585\varepsilon_{p}^{0.079} } \right)\left( {1 + 0.0412\ln \frac{{\dot{\varepsilon }}}{{\dot{\varepsilon }_{ref} }}} \right)\left[ {1 - \left( {\frac{{T - T_{ref} }}{{T_{m} - T_{ref} }}} \right)^{0.962} } \right] $$

**Figure 6:**
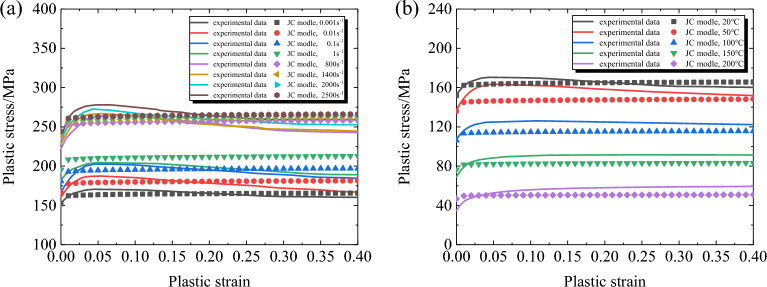
Comparisons between the experimental and the predicted stress by Johnson–Cook model. (**a**) *T* = 20°C;. (**b**) $\dot{\varepsilon }$=0.001s^−1^.

The comparison between the predicted results of the classical Johnson–Cook model and the experimental results for the 30% SGFR-PEEK is shown in Fig. 6. It is evident that the predicted values of the classical model are in good agreement with the experimental values at low strain rates and various temperatures. However, at high strain rates, there are significant discrepancies between the predicted and experimental values, and the classical model is unable to describe the material's deformation stages of work hardening, transition, and softening. This indicates that the classical Johnson–Cook model cannot fully reflect the mechanical behavior of the material over a wide range of strain rates and temperatures. To better describe the material's mechanical properties, it is necessary to improve the Johnson–Cook model.

To provide a more accurate description of the mechanical properties of materials, several studies have proposed methods to improve the JC model. These approaches often involve rewriting and refitting certain terms within the conventional JC model using polynomial or power functions, aiming to achieve better performance^21,22^. In this study, we followed the strategy outlined in these previous investigations and developed a modified JC model to better characterize the mechanical behavior of SGFR-PEEK composites.

Similarly, 0.001 s^−1^was used as the reference strain rate ${\dot{\varepsilon }}_{ref}$ and 23 °C as the reference temperature *T*_*ref*_ to determine the parameters associated with the modified Johnson–Cook model.

To enable the improved model to describe the behavior of materials such as process hardening, the first term (*A* + *Bε*^*n*^) in the classical Johnson–Cook model is rewritten in polynomial form as:

10 $$ \sigma (\varepsilon_{p} ) = A + B_{1} \varepsilon_{p} + B_{2} \varepsilon_{p}^{2} + B_{3} \varepsilon_{p}^{3} + B_{4} \varepsilon_{p}^{4} + B_{5} \varepsilon_{p}^{5} + B_{6} \varepsilon_{p}^{6} $$

The fitted values of *A* and *B*_1_ ~ *B*_6_ are given in Table 3.

**Table 3:** Values of *A* and *B*_1_ ~ *B*_6_ in $\sigma ({\varepsilon }_{p})$.

*A*	*B*_1_	*B*_2_	*B*_3_	*B*_4_	*B*_5_	*B*_6_
152.08	723.11	− 8932.65	47,939.41	− 130,099.27	173,933.63	− 90,815.68

To express the influence of strain rate on material properties, a function is constructed to fit the relationship between *σ*/*σ*(*ε*_*p*_) and ln($\dot{\varepsilon }$/${\dot{\varepsilon }}_{ref}$).

11 $$ \frac{\sigma }{{\sigma (\varepsilon_{p} )}} = 1 + C_{1} \ln \frac{{\dot{\varepsilon }}}{{\dot{\varepsilon }_{ref} }} + C_{2} \left( {\ln \frac{{\dot{\varepsilon }}}{{\dot{\varepsilon }_{ref} }}} \right)^{2} $$

The fitted curve in the figure is the average fitted line, which determines the values of *C*_1_, *C*_2_ in Eq. (11) as 0.02616 and 8.79 × 10^–4^.

To determine the influence of temperature on material properties, the relationship between $\mathrm{ln}(\sigma /\sigma ({\varepsilon }_{p}))$ and $\mathrm{ln}\left[(T-{T}_{ref})/({T}_{m}-{T}_{ref})\right]$ can be established according to Eq. (12).

12 $$ \ln \left[ {1 - \frac{\sigma }{{\sigma (\varepsilon_{p} )}}} \right] = m\ln \left( {\frac{{T - T_{ref} }}{{T_{m} - T_{ref} }}} \right) $$

The value of *m* can be determined from the slope of the average fitted line as 0.969.

The improved Johnson–Cook model with all parameters determined by the above calculations can be written as

13 $$ \left\{ \begin{gathered} \sigma = \sigma (\varepsilon_{p} )\left( {1 + C_{1} \ln \frac{{\dot{\varepsilon }}}{{\dot{\varepsilon }_{ref} }} + C_{2} \left( {\ln \frac{{\dot{\varepsilon }}}{{\dot{\varepsilon }_{ref} }}} \right)^{2} } \right)\left[ {1 - \left( {\frac{{T - T_{ref} }}{{T_{m} - T_{ref} }}} \right)^{m} } \right] \hfill \\ \sigma (\varepsilon_{p} ) = A + B_{1} \varepsilon_{p} + B_{2} \varepsilon_{p}^{2} + B_{3} \varepsilon_{p}^{3} + B_{4} \varepsilon_{p}^{4} + B_{5} \varepsilon_{p}^{5} + B_{6} \varepsilon_{p}^{6} \hfill \\ \end{gathered} \right. $$

**Figure 7:**
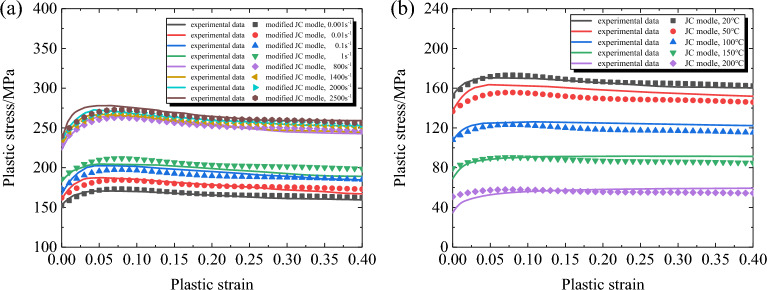
Comparisons between the experimental and the predicted stress by modified Johnson–Cook model. (**a**) *T* = 20°C;. (**b**) $\dot{\varepsilon }$=0.001s^−1^.

The comparison between the predicted results and the experimental results of the improved Johnson–Cook model is shown in Fig. 7. Compared with the classical Johnson–Cook model, the accuracy of the modified Johnson–Cook model in predicting the flow stress of the material at high strain rates has been significantly improved, and it can effectively describe the work hardening stage, transition stage, and softening stage of the material during deformation. By comparing the predicted values and experimental values at different strain rates and temperatures, it can be seen that the modified Johnson–Cook model can well describe the mechanical properties of the material.

### Constitutive equations considering fiber content

Based on the previous analysis, it can be observed that the modified Johnson–Cook model provides a more accurate description of the mechanical properties of 30% SGFR-PEEK compared to the classical Johnson–Cook model. To enhance the applicability of the constitutive model, it is worthwhile to consider the influence of fiber content. Following the fitting method applied to the modified Johnson–Cook model for 30% SGFR-PEEK in the previous section, the stress–strain curves of PEEK, 10% SGFR-PEEK, and 20% SGFR-PEEK were similarly fitted. The parameters of the modified Johnson–Cook model obtained for each case are presented in Table 4.

**Table 4:** The values of each parameter in the modified Johnson–Cook model.

	PEEK	10%SGFR-PEEK	20%SGFR-PEEK	30%SGFR-PEEK
*A*	134.18	138.75	146.28	152.08
*B*_1_	43.18	791.65	769.826	723.11
*B*_2_	− 680.68	− 16,217.99	− 11,385.05	− 8932.65
*B*_3_	− 1824.16	148,880.91	73,855.43	47,939.41
*B*_4_	48,495.76	− 689,529.17	− 245,033.29	− 130,099.27
*B*_5_	− 191,474.18	1,554,870	403,452.91	173,933.63
*B*_6_	227,303.85	− 1,354,000	− 260,496.28	− 90,815.68
*C*_1_	0.02683	0.02542	0.02573	0.02616
*C*_2_	9.88 × 10^–4^	10.26 × 10^–4^	9.41 × 10^–4^	8.79 × 10^–4^
*m*	0.969	0.971	0.967	0.969

Table 4 reveals distinct characteristics of various parameters in the modified Johnson–Cook model as a function of the fiberglass mass fraction. Specifically, the parameter *A* exhibits a linear growth trend with an increase in mass fraction. The relationship between *A* and the mass fraction *w*, as derived from linear fitting, can be expressed as follows:

14 $$ A(w) = 134.18 + 58.91\frac{w}{100\% } $$

The specific values of *B*_1_ to *B*_6_ in the constitutive model at different mass fractions do not exhibit evident regularities. Parameters representing strain rate effects (*C*_1_, *C*_2_) and those reflecting temperature effects (*m*) have values that are relatively close across various models. This suggests that changes in fiberglass content alter the specific characteristics of the stress–strain curve but do not significantly impact the material's patterns of strain rate strengthening and temperature softening. Therefore, in practical applications, to obtain an accurate stress–strain relationship for the material, it is necessary to experimentally determine the specific values of *B*_1_ to *B*_6_. Meanwhile, the specific values of *A*, *C*_1_, *C*_2_, and *m* can be referenced from the computational results presented in this paper.

Following the above analysis, the modified Johnson–Cook model for SGFR-PEEK composites, considering fiber content, can be expressed as follows:

15 $$ \left\{ {\begin{array}{*{20}l} {\sigma = \sigma (\varepsilon_{p} )\left( {1 + C_{1} \ln \frac{{\dot{\varepsilon }}}{{\dot{\varepsilon }_{ref} }} + C_{2} \left( {\ln \frac{{\dot{\varepsilon }}}{{\dot{\varepsilon }_{ref} }}} \right)^{2} } \right)\left[ {1 - \left( {\frac{{T - T_{ref} }}{{T_{m} - T_{ref} }}} \right)^{m} } \right]} \hfill \\ {\sigma (\varepsilon_{p} ) = A(w) + B_{1} \varepsilon_{p} + B_{2} \varepsilon_{p}^{2} + B_{3} \varepsilon_{p}^{3} + B_{4} \varepsilon_{p}^{4} + B_{5} \varepsilon_{p}^{5} + B_{6} \varepsilon_{p}^{6} } \hfill \\ {A(w) = 134.18 + 58.91\frac{w}{100\% }} \hfill \\ \end{array} } \right. $$

where *w* represents the mass fraction of fiberglass, the values of *B*_1_ to *B*_6_ can be experimentally determined. Additionally, *C*_1_ = 0.02604, *C*_2_ = 9.58 × 10^–4^, *m* = 0.969.

Table 5 illustrates the error between the results calculated from Eq. (15) and experimental results. It can be observed that under conditions ranging from 23°C to 100°C, the error between calculated values and experimental results is generally within 10%. However, at temperatures of 150°C and 200°C, the model exhibits larger errors. This is attributed to the model sacrificing the prediction accuracy of yield stress to fit the overall curve. As the strain exceeds 0.3, the model's error decreases to within 10%.

**Table 5:** The error between the improved Johnson–Cook model and experimental results.

*T*/°C	$\dot{\varepsilon }$/s^−1^	PEEK (%)	10%SGFR-PEEK (%)	20%SGFR-PEEK (%)	30%SGFR-PEEK (%)
23	0.001	1.22	1.30	1.18	0.94
	0.01	1.18	1.99	3.65	1.07
	0.1	3.55	3.98	6.12	4.02
	1	5.12	5.08	4.46	2.30
	100	3.97			
	800				4.58
	1000		1.56	3.28	
	1200		5.26		
	1400			1.86	3.84
	2000		2.38	1.84	3.31
	2500		1.41		2.76
	2900	2.40			
50	0.001	1.94	1.96	1.93	2.13
100		6.80	6.83	7.12	6.97
150 ($\sigma_{{{\text{yield}}}}$)		26.43	29.18	29.44	26.52
150 (*ε* = 0.3)		3.24	2.17	1.15	2.53
200 ($\sigma_{{{\text{yield}}}}$)		56.12	90.10	84.12	90.03
200 (*ε* = 0.3)		2.12	1.49	2.51	1.75

## Finite element calculation

**Figure 8:**
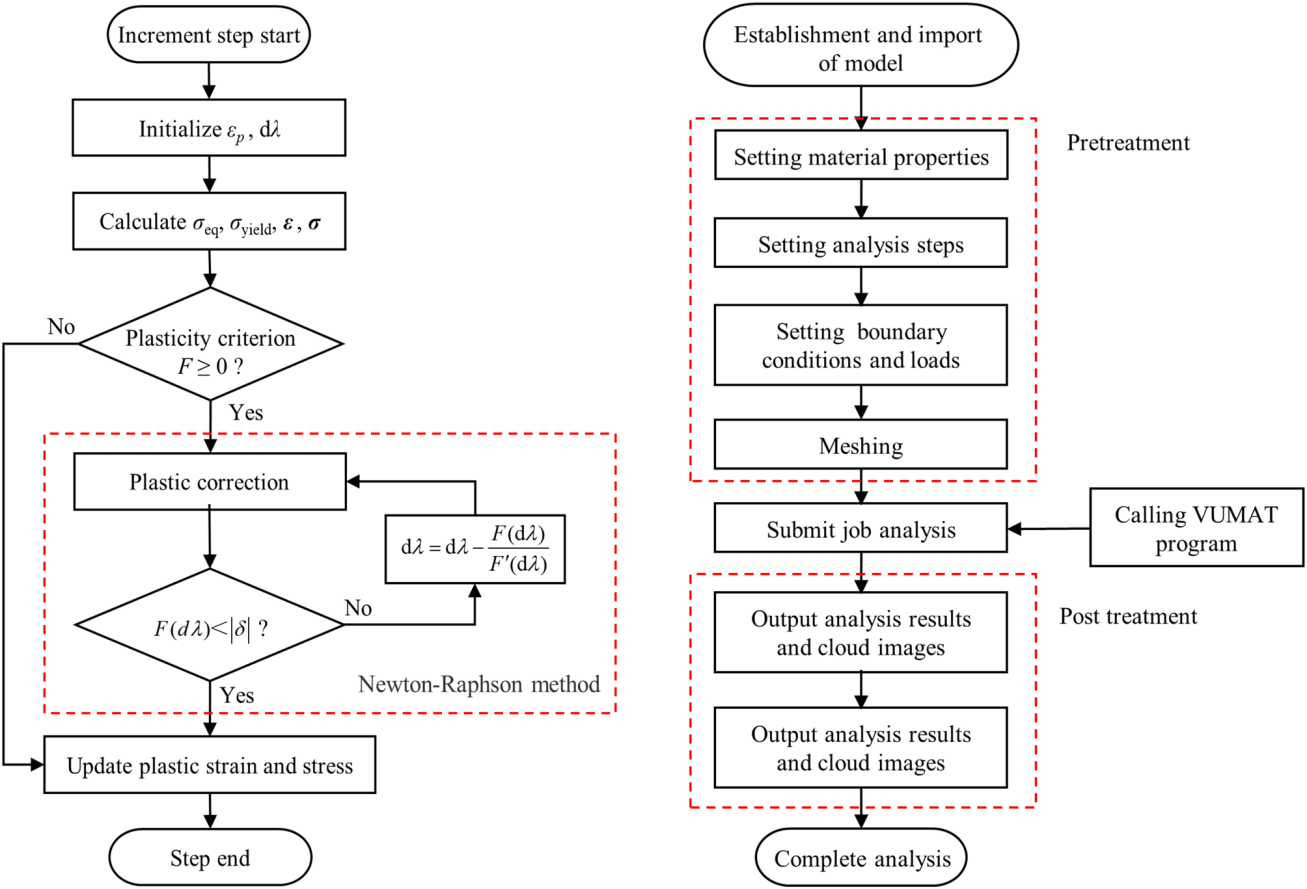
Flowchart of the VUMAT (left) and ABAQUS analysis process (right).

In order to achieve more accurate numerical calculations of SGFR-PEEK composites under various loading conditions to assist engineering practice, a user-defined material subroutine VUMAT was developed based on the modified Johnson–Cook model. The VUMAT subroutine can be used for explicit dynamic calculations, and its fixed-format interface ensures that it can be called by the ABAQUS main program. The flowchart of the VUMAT and ABAQUS analysis process is shown in Fig. 8.

In the subroutine VUMAT:

- Yield function: $$F = \sigma_{eq} - \sigma_{yield}$$.
- Flow rule: $${\text{d}}\varepsilon_{ij}^{p} = {\text{d}}\lambda \frac{{\partial \sigma_{eq} }}{{\partial \sigma_{ij} }}$$.
- Hardening law (see “Constitutive equations for SGFR + PEEK composites” section): $\sigma = \sigma (\varepsilon_{p} )(1 + C_{1} \ln \frac{{\dot{\varepsilon }}}{{\dot{\varepsilon }_{ref} }} + C_{2} (\ln \frac{{\dot{\varepsilon }}}{{\dot{\varepsilon }_{ref} }})^{2} )[1 - (\frac{{T - T_{ref} }}{{T_{m} - T_{ref} }})^{m} ]$.

Where *σ*_*eq*_ is equivalent stress, *σ*_*yeild*_ is yield Stress, *ε*_*ij*_ is plastic strain components, *σ*_*ij*_ is stress components, d*λ* is flow increment^32,33^.

**Figure 9:**
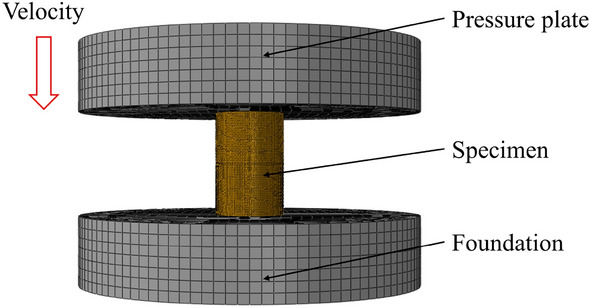
Finite element model for ABAQUS.

The VUMAT subroutine includes the parameters required by the improved Johnson–Cook model, as well as the material's elastic modulus and Poisson's ratio, which are 2800 MPa and 0.31, respectively. This paper established a finite element model consistent with the quasi-static experimental dimensions, as shown in Fig. 9. The material parameters of the specimen were defined by the VUMAT subroutine, while the compression platens and base were made of steel with an elastic modulus of 210 GPa and Poisson's ratio of 0.3, without considering their plastic deformation. The friction coefficient between the specimen and the platens is 0.2. During the simulation, the base was fixed, and the compression platens moved downward with a velocity set according to the experimental reference. The model mesh used eight-node hexahedral reduced integration elements (C3D8R), and the mesh of the specimen was refined, resulting in a total of 94,800 elements.

**Figure 10:**
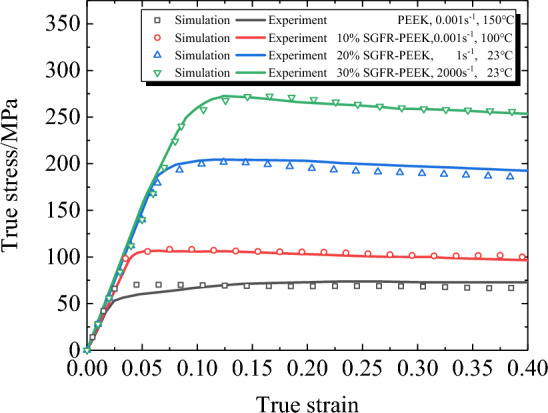
Comparison between the numerical results and experimental results.

The stress–strain curves of SGFR-PEEK composites with different mass fractions obtained from numerical simulations and experiments under various conditions are illustrated in Fig. 10. It is evident that within the plastic range, the results of numerical simulations under different conditions closely align with experimental outcomes. This indicates that users can simulate the mechanical properties of SGFR-PEEK composites with varying component mass fractions by defining necessary parameters in VUMAT. Furthermore, more sophisticated simulation calculations can be conducted to assist in simulation design.

**Figure 11:**
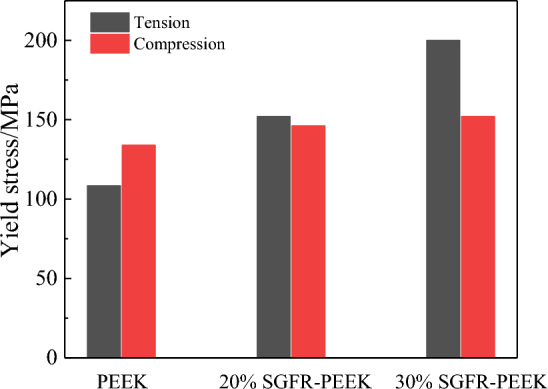
The yield stress in tension/compression of SGFR-PEEK composites.

Finally, it should be noted that, referring to the tensile properties of the material in various literature sources^9,34,35^, as illustrated in Fig. 11, the material exhibits different yield strengths during tension and compression. This implies that, despite the modified JC model and VUMAT subroutine have shown good performance in describing the compression performance of materials, significant deviations are still observed when characterizing the material under tensile stress. Achieving a comprehensive understanding of the material's tensile and compressive performance requires further experimentation and analysis in subsequent studies.

## Conclusion

Through quasi-static tests at different temperatures and Split Hopkinson Pressure Bar (SHPB) compression tests at room temperature, the mechanical properties of short glass fiber-reinforced poly-ether-ether-ketone (SGFR-PEEK) composites with varying mass fractions were investigated under different temperatures and strain rates. The results indicate that the yield stress of the material increases with an augmentation in the glass fiber content, demonstrating sensitivity to both temperature and strain rate. Specifically, the yield stress decreases with an increase in temperature but increases with higher strain rates. A modified Johnson–Cook model was established based on experimental findings to describe the material's mechanical behavior. This model incorporates glass fiber mass fraction and effectively captures the dependency of the material's mechanical performance on temperature and strain rate. Furthermore, the enhanced constitutive model was implemented in ABAQUS software using the VUMAT subroutine to simulate SGFR-PEEK composite behavior. The accuracy of the developed constitutive model was validated through a comparison of simulation results with experimental data. The research findings contribute valuable insights for the engineering applications and numerical simulations of SGFR-PEEK composites.

## Data Availability

The datasets used and/or analysed during the current study are available from the corresponding author on reasonable request.
